# 3,3′,5,5′-Tetra-*tert*-butyl-2′-hy­droxy-[1,1′-biphen­yl]-2-yl 4-methyl­benzene­sulfonate

**DOI:** 10.1107/S1600536812051938

**Published:** 2013-01-04

**Authors:** Chunli Jian, Jinjin Zhang, Lei Wang, Ning Tang, Jincai Wu

**Affiliations:** a Department of Chemistry, Lanzhou University, Lanzhou 730000, People’s Republic of China

## Abstract

In the title mol­ecule, C_37_H_48_O_4_S, the benzene rings in the biphenyl fragment are inclined to each other at 61.1 (1)°. The hy­droxy group is involved in a weak intra­molecular O—H⋯O_sulfonate_ hydrogen bond. One *tert*-butyl group is disodered over two orientations in a 0.682 (17):0.318 (17) ratio. In the crystal, weak C—H⋯O hydrogen bonds link the mol­ecules into columns in direction [100].

## Related literature

For applications of coordination complexes with close ligands in the ring-opening polymerization of cyclic esters, see: Wu *et al.* (2006 ▶). For the crystal structures of related compounds, see: Wu *et al.* (2009 ▶); Wang & Wu (2012 ▶).
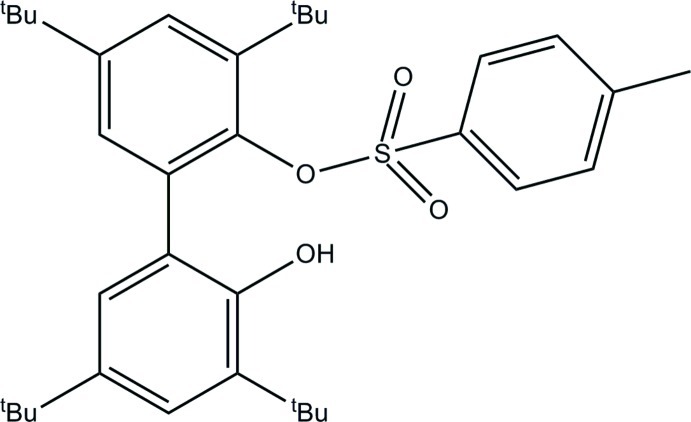


## Experimental

### 

#### Crystal data

C_35_H_48_O_4_S
*M* *_r_* = 564.79
Triclinic, 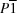

*a* = 9.885 (2) Å
*b* = 12.948 (3) Å
*c* = 13.600 (3) Å
α = 101.761 (2)°
β = 102.539 (2)°
γ = 92.004 (2)°
*V* = 1657.8 (6) Å^3^
*Z* = 2
Mo *K*α radiation
μ = 0.13 mm^−1^
*T* = 296 K
0.28 × 0.22 × 0.21 mm

#### Data collection

Bruker SMART APEXII diffractometer
Absorption correction: multi-scan (*SADABS*; Bruker, 2004) ▶ *T*_min_ = 0.965, *T*_max_ = 0.973
11516 measured reflections
7609 independent reflections
3453 reflections with *I* > 2σ(*I*)
*R*_int_ = 0.036

#### Refinement

*R*[*F* ^2^ > 2σ(*F* ^2^)] = 0.074
*wR*(*F* ^2^) = 0.205
*S* = 0.99
7609 reflections
389 parameters
H-atom parameters constrained
Δρ_max_ = 0.30 e Å^−3^
Δρ_min_ = −0.28 e Å^−3^

### 

Data collection: *APEX2* (Bruker, 2004 ▶); cell refinement: *SAINT* (Bruker, 2004 ▶); data reduction: *SAINT*; program(s) used to solve structure: *SHELXS97* (Sheldrick, 2008 ▶); program(s) used to refine structure: *SHELXL97* (Sheldrick, 2008 ▶); molecular graphics: *SHELXTL* (Sheldrick, 2008 ▶); software used to prepare material for publication: *SHELXTL*.

## Supplementary Material

Crystal structure: contains datablock(s) I, global. DOI: 10.1107/S1600536812051938/cv5377sup1.cif

Structure factors: contains datablock(s) I. DOI: 10.1107/S1600536812051938/cv5377Isup2.hkl

Supplementary material file. DOI: 10.1107/S1600536812051938/cv5377Isup3.cml

Additional supplementary materials:  crystallographic information; 3D view; checkCIF report

## Figures and Tables

**Table 1:** Hydrogen-bond geometry (Å, °)

*D*—H⋯*A*	*D*—H	H⋯*A*	*D*⋯*A*	*D*—H⋯*A*
O2—H2*A*⋯O4	0.82	2.59	3.322 (4)	149
C24—H24*C*⋯O3^i^	0.96	2.67	3.372 (4)	130
C25—H25*C*⋯O4^ii^	0.96	2.64	3.500 (4)	150
C20—H20*B*⋯O3^ii^	0.96	2.69	3.609 (5)	160

Symmetry codes: (i) 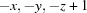; (ii) 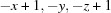.

**Fig. 1.**
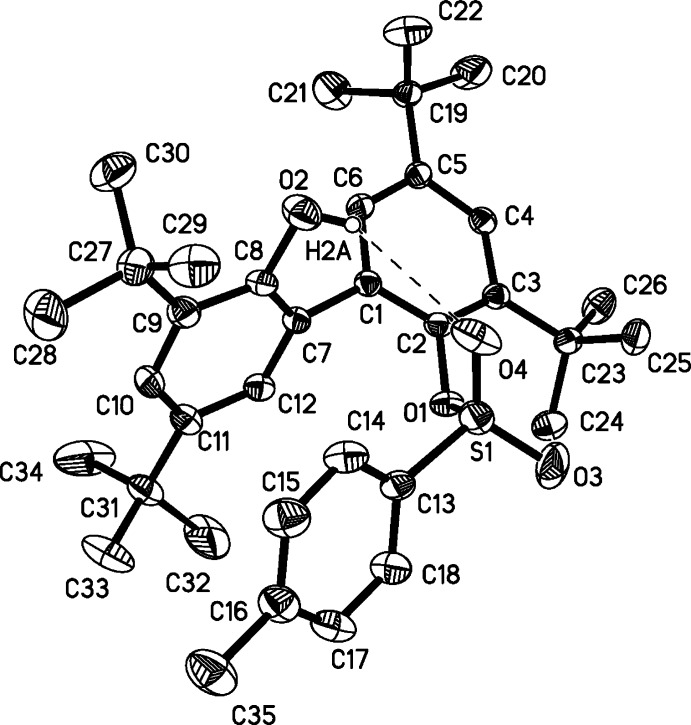
Molecular structure of (I) showing the atomic numbering and 30% probability displacement ellipsoids. C-bound H atoms were omitted for clarity. For the disordered tert-butyl group, only major part is shown. Dashed line denotes hydrogen bond.

## supplementary crystallographic information

### Comment

Polyesters as potentially valuable "green plastics" are attracting increasing attention. Many coordination complexes have been designed and synthesized as initiators or catalysts for the ring-opening polymerization of cyclic esters (Wu *et al.*, 2006). Herewith we present the title compound (I).

In (I) (Fig. 1), all bond lengths and angles are normal and correspond to those observed in the related compounds (Wu *et al.*, 2009; Wang *et al.*, 2012). Two benzene rings in the biphenyl fragment are inclined to each other at 61.1 (1)°. The hydroxy group is involved in a weak intramolecular O2—H2A···O4 hydrogen bond (Table 1). In the crystal, weak intermolecular C—H···O hydrogen bonds (Table 1) link the molecules into columns in [100].

### Experimental

This title compound was synthesized by condensation of 3,3',5,5'-tetra-*tert*-butyl-[1,1'-biphenyl]-2,2'-diol and and 4- methylbenzene-1-sulfonyl chloride in dichloromethane at 0°C for about 1 h in the presence of 10 equiv. triethylamine. The crystals were obtained by slow cooling a hexane solution.

### Refinement

C-bound H atoms were geometrically positioned, and were treated as riding on their parent atoms, with C—H= 0.93–0.96 Å and U_iso_(H) = 1.2-1.5 U_eq_(C). The O-bound H atom was located in a difference Fourier map, but placed in idealized position (O—H = 0.82 Å), and refined as riding, with U_iso_(H) = 1.5 U_eq_(O).

### Crystal data

**Table:**

C_35_H_48_O_4_S	*Z* = 2
*M**_r_* = 564.79	*F*(000) = 612
Triclinic, *P*1	*D*_x_ = 1.131 Mg m^−^^3^
*a* = 9.885 (2) Å	Mo *K*α radiation, λ = 0.71073 Å
*b* = 12.948 (3) Å	Cell parameters from 1685 reflections
*c* = 13.600 (3) Å	θ = 2.3–23.6°
α = 101.761 (2)°	µ = 0.13 mm^−^^1^
β = 102.539 (2)°	*T* = 296 K
γ = 92.004 (2)°	Block, colourless
*V* = 1657.8 (6) Å^3^	0.28 × 0.22 × 0.21 mm

### Data collection

**Table:**

Bruker SMART APEXII diffractometer	7609 independent reflections
Radiation source: fine-focus sealed tube	3453 reflections with *I* > 2σ(*I*)
Graphite monochromator	*R*_int_ = 0.036
phi and ω scans	θ_max_ = 28.3°, θ_min_ = 2.3°
Absorption correction: multi-scan (*SADABS*; Bruker, 2004)	*h* = −12→12
*T*_min_ = 0.965, *T*_max_ = 0.973	*k* = −17→12
11516 measured reflections	*l* = −17→18

### Refinement

**Table:**

Refinement on *F*^2^	Primary atom site location: structure-invariant direct methods
Least-squares matrix: full	Secondary atom site location: difference Fourier map
*R*[*F*^2^ > 2σ(*F*^2^)] = 0.074	Hydrogen site location: inferred from neighbouring sites
*wR*(*F*^2^) = 0.205	H-atom parameters constrained
*S* = 0.99	*w* = 1/[σ^2^(*F*_o_^2^) + (0.0909*P*)^2^] where *P* = (*F*_o_^2^ + 2*F*_c_^2^)/3
7609 reflections	(Δ/σ)_max_ = 0.001
389 parameters	Δρ_max_ = 0.30 e Å^−^^3^
0 restraints	Δρ_min_ = −0.28 e Å^−^^3^

### Special details

**Table:**

Geometry. All e.s.d.'s (except the e.s.d. in the dihedral angle between two l.s. planes) are estimated using the full covariance matrix. The cell e.s.d.'s are taken into account individually in the estimation of e.s.d.'s in distances, angles and torsion angles; correlations between e.s.d.'s in cell parameters are only used when they are defined by crystal symmetry. An approximate (isotropic) treatment of cell e.s.d.'s is used for estimating e.s.d.'s involving l.s. planes.
Refinement. Refinement of *F*^2^ against ALL reflections. The weighted *R*-factor *wR* and goodness of fit *S* are based on *F*^2^, conventional *R*-factors *R* are based on *F*, with *F* set to zero for negative *F*^2^. The threshold expression of *F*^2^ > σ(*F*^2^) is used only for calculating *R*-factors(gt) *etc*. and is not relevant to the choice of reflections for refinement. *R*-factors based on *F*^2^ are statistically about twice as large as those based on *F*, and *R*- factors based on ALL data will be even larger.

### Fractional atomic coordinates and isotropic or equivalent isotropic displacement parameters (Å^2^)

**Table:**

	*x*	*y*	*z*	*U*_iso_*/*U*_eq_	Occ. (<1)
S1	0.22545 (10)	0.17764 (8)	0.44203 (7)	0.0562 (3)	
O1	0.1844 (2)	0.07858 (17)	0.34604 (16)	0.0449 (6)	
O2	0.5120 (2)	0.26901 (19)	0.27643 (19)	0.0600 (7)	
H2A	0.5103	0.2567	0.3330	0.090*	
O3	0.1712 (3)	0.1483 (3)	0.5218 (2)	0.0948 (11)	
O4	0.3688 (3)	0.2077 (2)	0.45884 (19)	0.0732 (8)	
C1	0.3461 (3)	0.0734 (2)	0.2373 (2)	0.0337 (7)	
C2	0.2906 (3)	0.0266 (2)	0.3045 (2)	0.0354 (7)	
C3	0.3277 (3)	−0.0710 (2)	0.3269 (2)	0.0350 (7)	
C4	0.4364 (3)	−0.1131 (2)	0.2843 (2)	0.0370 (7)	
H4B	0.4663	−0.1768	0.2988	0.044*	
C5	0.5024 (3)	−0.0665 (2)	0.2222 (2)	0.0364 (7)	
C6	0.4537 (3)	0.0270 (2)	0.1985 (2)	0.0373 (7)	
H6A	0.4942	0.0592	0.1556	0.045*	
C7	0.2913 (3)	0.1695 (2)	0.2016 (2)	0.0348 (7)	
C8	0.3770 (3)	0.2606 (3)	0.2169 (2)	0.0379 (8)	
C9	0.3317 (3)	0.3460 (3)	0.1729 (2)	0.0439 (8)	
C10	0.1955 (3)	0.3340 (3)	0.1159 (2)	0.0439 (8)	
H10A	0.1629	0.3895	0.0860	0.053*	
C11	0.1041 (3)	0.2450 (3)	0.1002 (2)	0.0425 (8)	
C12	0.1548 (3)	0.1631 (3)	0.1449 (2)	0.0396 (8)	
H12A	0.0965	0.1026	0.1368	0.047*	
C13	0.1268 (4)	0.2766 (3)	0.4004 (2)	0.0454 (9)	
C14	0.1954 (4)	0.3687 (3)	0.3934 (3)	0.0603 (10)	
H14A	0.2915	0.3746	0.4020	0.072*	
C15	0.1179 (4)	0.4518 (3)	0.3734 (3)	0.0672 (11)	
H15A	0.1641	0.5142	0.3696	0.081*	
C16	−0.0226 (4)	0.4467 (3)	0.3590 (3)	0.0572 (10)	
C17	−0.0889 (4)	0.3533 (3)	0.3653 (3)	0.0654 (11)	
H17A	−0.1852	0.3474	0.3553	0.078*	
C18	−0.0154 (4)	0.2684 (3)	0.3861 (3)	0.0596 (10)	
H18A	−0.0618	0.2062	0.3903	0.072*	
C19	0.6240 (3)	−0.1130 (3)	0.1791 (2)	0.0417 (8)	
C20	0.6596 (4)	−0.2182 (3)	0.2076 (4)	0.0798 (13)	
H20A	0.5811	−0.2694	0.1788	0.120*	
H20B	0.6826	−0.2090	0.2813	0.120*	
H20C	0.7376	−0.2427	0.1807	0.120*	
C21	0.5913 (5)	−0.1263 (4)	0.0628 (3)	0.0905 (16)	
H21A	0.5101	−0.1746	0.0326	0.136*	
H21B	0.6686	−0.1539	0.0368	0.136*	
H21C	0.5745	−0.0590	0.0454	0.136*	
C22	0.7542 (4)	−0.0359 (3)	0.2265 (3)	0.0781 (13)	
H22A	0.7760	−0.0275	0.3002	0.117*	
H22B	0.7372	0.0315	0.2093	0.117*	
H22C	0.8310	−0.0635	0.1998	0.117*	
C23	0.2605 (3)	−0.1305 (3)	0.3933 (2)	0.0405 (8)	
C24	0.1016 (3)	−0.1278 (3)	0.3695 (3)	0.0634 (11)	
H24A	0.0636	−0.1592	0.2979	0.095*	
H24B	0.0784	−0.0558	0.3840	0.095*	
H24C	0.0635	−0.1668	0.4115	0.095*	
C25	0.3219 (4)	−0.0820 (3)	0.5080 (2)	0.0553 (10)	
H25A	0.2796	−0.1190	0.5495	0.083*	
H25B	0.3041	−0.0087	0.5227	0.083*	
H25C	0.4204	−0.0879	0.5235	0.083*	
C26	0.2909 (4)	−0.2467 (3)	0.3749 (3)	0.0616 (10)	
H26A	0.2526	−0.2800	0.3039	0.092*	
H26B	0.2496	−0.2815	0.4184	0.092*	
H26C	0.3896	−0.2515	0.3907	0.092*	
C27	0.4274 (4)	0.4459 (3)	0.1849 (3)	0.0552 (10)	
C28	0.3500 (5)	0.5268 (3)	0.1297 (4)	0.0913 (15)	
H28A	0.2729	0.5475	0.1594	0.137*	
H28B	0.3165	0.4953	0.0577	0.137*	
H28C	0.4124	0.5879	0.1375	0.137*	
C29	0.4797 (4)	0.5009 (3)	0.2986 (3)	0.0705 (12)	
H29A	0.5289	0.4528	0.3357	0.106*	
H29B	0.4020	0.5224	0.3274	0.106*	
H29C	0.5410	0.5620	0.3041	0.106*	
C30	0.5501 (4)	0.4157 (3)	0.1359 (3)	0.0760 (12)	
H30A	0.6003	0.3654	0.1692	0.114*	
H30B	0.6109	0.4779	0.1440	0.114*	
H30C	0.5161	0.3850	0.0638	0.114*	
C31	−0.0447 (3)	0.2368 (3)	0.0343 (3)	0.0540 (10)	
C32	−0.1409 (7)	0.1544 (10)	0.0610 (11)	0.117 (6)	0.682 (17)
H32A	−0.1383	0.0861	0.0181	0.175*	0.682 (17)
H32B	−0.2344	0.1750	0.0491	0.175*	0.682 (17)
H32C	−0.1094	0.1514	0.1322	0.175*	0.682 (17)
C33	−0.1088 (11)	0.3421 (8)	0.0594 (10)	0.096 (4)	0.682 (17)
H33A	−0.0511	0.3979	0.0485	0.143*	0.682 (17)
H33B	−0.1158	0.3565	0.1301	0.143*	0.682 (17)
H33C	−0.1999	0.3380	0.0153	0.143*	0.682 (17)
C34	−0.0426 (8)	0.2085 (15)	−0.0744 (5)	0.133 (8)	0.682 (17)
H34A	0.0150	0.2610	−0.0905	0.200*	0.682 (17)
H34B	−0.1355	0.2051	−0.1155	0.200*	0.682 (17)
H34C	−0.0060	0.1408	−0.0890	0.200*	0.682 (17)
C32'	−0.083 (2)	0.1294 (14)	−0.036 (2)	0.104 (10)	0.318 (17)
H32D	−0.0401	0.1245	−0.0931	0.156*	0.318 (17)
H32E	−0.1822	0.1188	−0.0601	0.156*	0.318 (17)
H32F	−0.0512	0.0762	0.0019	0.156*	0.318 (17)
C33'	−0.048 (3)	0.315 (2)	−0.045 (3)	0.19 (2)	0.318 (17)
H33D	0.0433	0.3497	−0.0338	0.285*	0.318 (17)
H33E	−0.1127	0.3668	−0.0330	0.285*	0.318 (17)
H33F	−0.0756	0.2744	−0.1144	0.285*	0.318 (17)
C34'	−0.139 (2)	0.270 (4)	0.096 (3)	0.24 (3)	0.318 (17)
H34D	−0.1181	0.3437	0.1272	0.354*	0.318 (17)
H34E	−0.1323	0.2296	0.1482	0.354*	0.318 (17)
H34F	−0.2321	0.2589	0.0532	0.354*	0.318 (17)
C35	−0.1038 (5)	0.5394 (3)	0.3377 (3)	0.0850 (14)	
H35A	−0.0409	0.5973	0.3369	0.128*	
H35B	−0.1538	0.5608	0.3906	0.128*	
H35C	−0.1684	0.5190	0.2719	0.128*	

### Atomic displacement parameters (Å^2^)

**Table:**

	*U*^11^	*U*^22^	*U*^33^	*U*^12^	*U*^13^	*U*^23^
S1	0.0638 (7)	0.0656 (7)	0.0409 (5)	0.0307 (5)	0.0173 (4)	0.0055 (4)
O1	0.0382 (13)	0.0497 (14)	0.0511 (13)	0.0144 (11)	0.0198 (10)	0.0089 (11)
O2	0.0435 (15)	0.0594 (16)	0.0722 (17)	0.0024 (12)	0.0002 (12)	0.0173 (13)
O3	0.145 (3)	0.111 (2)	0.0595 (16)	0.073 (2)	0.0616 (18)	0.0397 (17)
O4	0.0506 (17)	0.077 (2)	0.0697 (17)	0.0227 (14)	−0.0067 (13)	−0.0153 (15)
C1	0.0331 (17)	0.0364 (18)	0.0316 (16)	0.0068 (14)	0.0079 (13)	0.0064 (14)
C2	0.0300 (17)	0.0417 (19)	0.0336 (16)	0.0078 (14)	0.0080 (13)	0.0047 (14)
C3	0.0322 (17)	0.0376 (18)	0.0339 (16)	0.0025 (14)	0.0072 (13)	0.0049 (14)
C4	0.0379 (18)	0.0336 (18)	0.0397 (17)	0.0075 (14)	0.0083 (14)	0.0081 (14)
C5	0.0386 (18)	0.0387 (19)	0.0331 (16)	0.0076 (15)	0.0109 (14)	0.0070 (14)
C6	0.0385 (18)	0.0404 (19)	0.0359 (16)	0.0077 (15)	0.0139 (14)	0.0087 (14)
C7	0.0343 (18)	0.0357 (19)	0.0363 (17)	0.0089 (15)	0.0103 (14)	0.0088 (14)
C8	0.0279 (17)	0.041 (2)	0.0448 (18)	0.0060 (15)	0.0077 (14)	0.0089 (15)
C9	0.045 (2)	0.037 (2)	0.052 (2)	0.0086 (16)	0.0145 (16)	0.0104 (16)
C10	0.047 (2)	0.039 (2)	0.050 (2)	0.0138 (17)	0.0115 (16)	0.0169 (16)
C11	0.042 (2)	0.041 (2)	0.0411 (18)	0.0096 (17)	0.0042 (15)	0.0045 (16)
C12	0.0364 (19)	0.0402 (19)	0.0414 (18)	0.0058 (15)	0.0102 (14)	0.0055 (15)
C13	0.047 (2)	0.049 (2)	0.0428 (19)	0.0156 (18)	0.0196 (16)	0.0045 (16)
C14	0.048 (2)	0.063 (3)	0.072 (3)	0.011 (2)	0.025 (2)	0.007 (2)
C15	0.069 (3)	0.050 (2)	0.084 (3)	0.011 (2)	0.027 (2)	0.006 (2)
C16	0.064 (3)	0.049 (2)	0.055 (2)	0.016 (2)	0.0137 (19)	0.0002 (19)
C17	0.041 (2)	0.075 (3)	0.077 (3)	0.017 (2)	0.0106 (19)	0.011 (2)
C18	0.048 (2)	0.061 (3)	0.071 (3)	0.012 (2)	0.0174 (19)	0.013 (2)
C19	0.043 (2)	0.042 (2)	0.0455 (19)	0.0149 (17)	0.0205 (16)	0.0098 (16)
C20	0.084 (3)	0.067 (3)	0.114 (3)	0.040 (2)	0.061 (3)	0.031 (3)
C21	0.095 (3)	0.135 (4)	0.047 (2)	0.063 (3)	0.030 (2)	0.008 (2)
C22	0.051 (3)	0.081 (3)	0.105 (3)	0.009 (2)	0.035 (2)	0.007 (3)
C23	0.0372 (19)	0.044 (2)	0.0434 (18)	0.0045 (16)	0.0141 (15)	0.0111 (16)
C24	0.043 (2)	0.079 (3)	0.075 (3)	−0.004 (2)	0.0185 (19)	0.027 (2)
C25	0.064 (2)	0.061 (2)	0.047 (2)	0.005 (2)	0.0170 (18)	0.0210 (18)
C26	0.077 (3)	0.047 (2)	0.069 (2)	−0.003 (2)	0.029 (2)	0.017 (2)
C27	0.055 (2)	0.038 (2)	0.077 (3)	−0.0006 (18)	0.018 (2)	0.020 (2)
C28	0.089 (3)	0.053 (3)	0.141 (4)	0.001 (2)	0.019 (3)	0.051 (3)
C29	0.065 (3)	0.047 (2)	0.093 (3)	−0.007 (2)	0.020 (2)	0.000 (2)
C30	0.064 (3)	0.077 (3)	0.094 (3)	−0.008 (2)	0.034 (2)	0.022 (3)
C31	0.040 (2)	0.062 (3)	0.054 (2)	0.0140 (19)	−0.0012 (17)	0.0087 (19)
C32	0.034 (4)	0.153 (10)	0.159 (13)	−0.020 (5)	−0.024 (6)	0.080 (9)
C33	0.055 (6)	0.093 (7)	0.130 (10)	0.036 (5)	0.000 (5)	0.023 (6)
C34	0.053 (5)	0.27 (2)	0.045 (4)	0.025 (9)	−0.004 (4)	−0.015 (8)
C32'	0.069 (13)	0.081 (13)	0.12 (2)	−0.010 (10)	−0.046 (14)	−0.005 (12)
C33'	0.17 (3)	0.14 (2)	0.20 (4)	−0.08 (2)	−0.14 (3)	0.12 (3)
C34'	0.040 (13)	0.43 (8)	0.14 (3)	0.06 (3)	−0.008 (14)	−0.13 (4)
C35	0.090 (3)	0.071 (3)	0.087 (3)	0.034 (3)	0.010 (3)	0.006 (3)

### Geometric parameters (Å, º)

**Table:**

S1—O4	1.413 (3)	C23—C26	1.524 (4)
S1—O3	1.419 (3)	C23—C25	1.536 (4)
S1—O1	1.602 (2)	C23—C24	1.537 (4)
S1—C13	1.748 (3)	C24—H24A	0.9600
O1—C2	1.424 (3)	C24—H24B	0.9600
O2—C8	1.390 (3)	C24—H24C	0.9600
O2—H2A	0.8200	C25—H25A	0.9600
C1—C2	1.387 (4)	C25—H25B	0.9600
C1—C6	1.390 (4)	C25—H25C	0.9600
C1—C7	1.502 (4)	C26—H26A	0.9600
C2—C3	1.402 (4)	C26—H26B	0.9600
C3—C4	1.403 (4)	C26—H26C	0.9600
C3—C23	1.537 (4)	C27—C30	1.531 (5)
C4—C5	1.387 (4)	C27—C29	1.532 (5)
C4—H4B	0.9300	C27—C28	1.540 (5)
C5—C6	1.391 (4)	C28—H28A	0.9600
C5—C19	1.537 (4)	C28—H28B	0.9600
C6—H6A	0.9300	C28—H28C	0.9600
C7—C8	1.381 (4)	C29—H29A	0.9600
C7—C12	1.392 (4)	C29—H29B	0.9600
C8—C9	1.404 (4)	C29—H29C	0.9600
C9—C10	1.385 (4)	C30—H30A	0.9600
C9—C27	1.535 (5)	C30—H30B	0.9600
C10—C11	1.390 (5)	C30—H30C	0.9600
C10—H10A	0.9300	C31—C34'	1.40 (2)
C11—C12	1.383 (4)	C31—C34	1.454 (8)
C11—C31	1.535 (4)	C31—C32'	1.499 (16)
C12—H12A	0.9300	C31—C33	1.531 (10)
C13—C18	1.374 (5)	C31—C32	1.552 (8)
C13—C14	1.382 (5)	C31—C33'	1.61 (2)
C14—C15	1.380 (5)	C32—H32A	0.9600
C14—H14A	0.9300	C32—H32B	0.9600
C15—C16	1.358 (5)	C32—H32C	0.9600
C15—H15A	0.9300	C33—H33A	0.9600
C16—C17	1.382 (5)	C33—H33B	0.9600
C16—C35	1.509 (5)	C33—H33C	0.9600
C17—C18	1.382 (5)	C34—H34A	0.9600
C17—H17A	0.9300	C34—H34B	0.9600
C18—H18A	0.9300	C34—H34C	0.9600
C19—C21	1.516 (5)	C32'—H32D	0.9600
C19—C20	1.523 (5)	C32'—H32E	0.9600
C19—C22	1.535 (5)	C32'—H32F	0.9600
C20—H20A	0.9600	C33'—H33D	0.9600
C20—H20B	0.9600	C33'—H33E	0.9600
C20—H20C	0.9600	C33'—H33F	0.9600
C21—H21A	0.9600	C34'—H34D	0.9600
C21—H21B	0.9600	C34'—H34E	0.9600
C21—H21C	0.9600	C34'—H34F	0.9600
C22—H22A	0.9600	C35—H35A	0.9600
C22—H22B	0.9600	C35—H35B	0.9600
C22—H22C	0.9600	C35—H35C	0.9600
			
O4—S1—O3	120.22 (19)	C23—C25—H25B	109.5
O4—S1—O1	108.52 (13)	H25A—C25—H25B	109.5
O3—S1—O1	105.68 (17)	C23—C25—H25C	109.5
O4—S1—C13	110.24 (18)	H25A—C25—H25C	109.5
O3—S1—C13	107.12 (16)	H25B—C25—H25C	109.5
O1—S1—C13	103.78 (14)	C23—C26—H26A	109.5
C2—O1—S1	119.80 (19)	C23—C26—H26B	109.5
C8—O2—H2A	109.5	H26A—C26—H26B	109.5
C2—C1—C6	118.4 (3)	C23—C26—H26C	109.5
C2—C1—C7	122.9 (2)	H26A—C26—H26C	109.5
C6—C1—C7	118.7 (3)	H26B—C26—H26C	109.5
C1—C2—C3	123.2 (3)	C30—C27—C29	110.4 (3)
C1—C2—O1	117.0 (3)	C30—C27—C9	109.2 (3)
C3—C2—O1	119.7 (3)	C29—C27—C9	111.1 (3)
C2—C3—C4	114.7 (3)	C30—C27—C28	107.8 (3)
C2—C3—C23	125.1 (2)	C29—C27—C28	107.1 (3)
C4—C3—C23	120.2 (3)	C9—C27—C28	111.1 (3)
C5—C4—C3	124.7 (3)	C27—C28—H28A	109.5
C5—C4—H4B	117.6	C27—C28—H28B	109.5
C3—C4—H4B	117.6	H28A—C28—H28B	109.5
C4—C5—C6	117.0 (3)	C27—C28—H28C	109.5
C4—C5—C19	123.6 (3)	H28A—C28—H28C	109.5
C6—C5—C19	119.5 (3)	H28B—C28—H28C	109.5
C1—C6—C5	121.7 (3)	C27—C29—H29A	109.5
C1—C6—H6A	119.2	C27—C29—H29B	109.5
C5—C6—H6A	119.2	H29A—C29—H29B	109.5
C8—C7—C12	119.6 (3)	C27—C29—H29C	109.5
C8—C7—C1	121.1 (3)	H29A—C29—H29C	109.5
C12—C7—C1	119.1 (3)	H29B—C29—H29C	109.5
C7—C8—O2	120.0 (3)	C27—C30—H30A	109.5
C7—C8—C9	121.6 (3)	C27—C30—H30B	109.5
O2—C8—C9	118.4 (3)	H30A—C30—H30B	109.5
C10—C9—C8	115.9 (3)	C27—C30—H30C	109.5
C10—C9—C27	121.8 (3)	H30A—C30—H30C	109.5
C8—C9—C27	122.3 (3)	H30B—C30—H30C	109.5
C9—C10—C11	124.8 (3)	C34'—C31—C34	138.6 (13)
C9—C10—H10A	117.6	C34'—C31—C32'	115 (2)
C11—C10—H10A	117.6	C34—C31—C32'	53.6 (9)
C12—C11—C10	116.7 (3)	C34'—C31—C33	48 (2)
C12—C11—C31	121.5 (3)	C34—C31—C33	110.9 (7)
C10—C11—C31	121.7 (3)	C32'—C31—C33	139.1 (8)
C11—C12—C7	121.3 (3)	C34'—C31—C11	111.3 (11)
C11—C12—H12A	119.3	C34—C31—C11	109.6 (4)
C7—C12—H12A	119.3	C32'—C31—C11	111.2 (7)
C18—C13—C14	120.2 (3)	C33—C31—C11	109.7 (5)
C18—C13—S1	120.8 (3)	C34'—C31—C32	59 (2)
C14—C13—S1	118.6 (3)	C34—C31—C32	110.2 (7)
C15—C14—C13	118.4 (4)	C32'—C31—C32	59.9 (11)
C15—C14—H14A	120.8	C33—C31—C32	104.9 (6)
C13—C14—H14A	120.8	C11—C31—C32	111.5 (4)
C16—C15—C14	123.0 (4)	C34'—C31—C33'	108 (2)
C16—C15—H15A	118.5	C34—C31—C33'	52.3 (12)
C14—C15—H15A	118.5	C32'—C31—C33'	103.5 (13)
C15—C16—C17	117.4 (4)	C33—C31—C33'	62.6 (15)
C15—C16—C35	121.5 (4)	C11—C31—C33'	108.0 (9)
C17—C16—C35	121.1 (4)	C32—C31—C33'	140.5 (9)
C16—C17—C18	121.6 (4)	C31—C32—H32A	109.5
C16—C17—H17A	119.2	C31—C32—H32B	109.5
C18—C17—H17A	119.2	H32A—C32—H32B	109.5
C13—C18—C17	119.4 (4)	C31—C32—H32C	109.5
C13—C18—H18A	120.3	H32A—C32—H32C	109.5
C17—C18—H18A	120.3	H32B—C32—H32C	109.5
C21—C19—C20	109.8 (3)	C31—C33—H33A	109.5
C21—C19—C22	109.0 (3)	C31—C33—H33B	109.5
C20—C19—C22	106.5 (3)	H33A—C33—H33B	109.5
C21—C19—C5	110.1 (3)	C31—C33—H33C	109.5
C20—C19—C5	112.8 (3)	H33A—C33—H33C	109.5
C22—C19—C5	108.5 (3)	H33B—C33—H33C	109.5
C19—C20—H20A	109.5	C31—C34—H34A	109.5
C19—C20—H20B	109.5	C31—C34—H34B	109.5
H20A—C20—H20B	109.5	H34A—C34—H34B	109.5
C19—C20—H20C	109.5	C31—C34—H34C	109.5
H20A—C20—H20C	109.5	H34A—C34—H34C	109.5
H20B—C20—H20C	109.5	H34B—C34—H34C	109.5
C19—C21—H21A	109.5	C31—C32'—H32D	109.5
C19—C21—H21B	109.5	C31—C32'—H32E	109.5
H21A—C21—H21B	109.5	H32D—C32'—H32E	109.5
C19—C21—H21C	109.5	C31—C32'—H32F	109.5
H21A—C21—H21C	109.5	H32D—C32'—H32F	109.5
H21B—C21—H21C	109.5	H32E—C32'—H32F	109.5
C19—C22—H22A	109.5	C31—C33'—H33D	109.5
C19—C22—H22B	109.5	C31—C33'—H33E	109.5
H22A—C22—H22B	109.5	H33D—C33'—H33E	109.5
C19—C22—H22C	109.5	C31—C33'—H33F	109.5
H22A—C22—H22C	109.5	H33D—C33'—H33F	109.5
H22B—C22—H22C	109.5	H33E—C33'—H33F	109.5
C26—C23—C25	107.1 (3)	C31—C34'—H34D	109.5
C26—C23—C3	111.2 (2)	C31—C34'—H34E	109.5
C25—C23—C3	109.5 (3)	H34D—C34'—H34E	109.5
C26—C23—C24	107.1 (3)	C31—C34'—H34F	109.5
C25—C23—C24	109.8 (3)	H34D—C34'—H34F	109.5
C3—C23—C24	112.0 (3)	H34E—C34'—H34F	109.5
C23—C24—H24A	109.5	C16—C35—H35A	109.5
C23—C24—H24B	109.5	C16—C35—H35B	109.5
H24A—C24—H24B	109.5	H35A—C35—H35B	109.5
C23—C24—H24C	109.5	C16—C35—H35C	109.5
H24A—C24—H24C	109.5	H35A—C35—H35C	109.5
H24B—C24—H24C	109.5	H35B—C35—H35C	109.5
C23—C25—H25A	109.5		
			
O4—S1—O1—C2	9.7 (3)	O4—S1—C13—C14	−2.6 (3)
O3—S1—O1—C2	−120.5 (2)	O3—S1—C13—C14	129.8 (3)
C13—S1—O1—C2	127.0 (2)	O1—S1—C13—C14	−118.7 (3)
C6—C1—C2—C3	−6.7 (4)	C18—C13—C14—C15	1.0 (5)
C7—C1—C2—C3	170.8 (3)	S1—C13—C14—C15	−171.8 (3)
C6—C1—C2—O1	177.5 (3)	C13—C14—C15—C16	−0.9 (6)
C7—C1—C2—O1	−4.9 (4)	C14—C15—C16—C17	0.2 (6)
S1—O1—C2—C1	−80.7 (3)	C14—C15—C16—C35	179.8 (4)
S1—O1—C2—C3	103.4 (3)	C15—C16—C17—C18	0.4 (6)
C1—C2—C3—C4	6.2 (4)	C35—C16—C17—C18	−179.2 (3)
O1—C2—C3—C4	−178.2 (3)	C14—C13—C18—C17	−0.4 (5)
C1—C2—C3—C23	−174.5 (3)	S1—C13—C18—C17	172.2 (3)
O1—C2—C3—C23	1.1 (4)	C16—C17—C18—C13	−0.3 (6)
C2—C3—C4—C5	−1.8 (4)	C4—C5—C19—C21	125.8 (4)
C23—C3—C4—C5	179.0 (3)	C6—C5—C19—C21	−54.0 (4)
C3—C4—C5—C6	−2.0 (5)	C4—C5—C19—C20	2.8 (5)
C3—C4—C5—C19	178.2 (3)	C6—C5—C19—C20	−177.0 (3)
C2—C1—C6—C5	2.5 (4)	C4—C5—C19—C22	−115.0 (4)
C7—C1—C6—C5	−175.1 (3)	C6—C5—C19—C22	65.2 (4)
C4—C5—C6—C1	1.6 (4)	C2—C3—C23—C26	162.0 (3)
C19—C5—C6—C1	−178.6 (3)	C4—C3—C23—C26	−18.8 (4)
C2—C1—C7—C8	123.0 (3)	C2—C3—C23—C25	−79.9 (4)
C6—C1—C7—C8	−59.5 (4)	C4—C3—C23—C25	99.3 (3)
C2—C1—C7—C12	−62.3 (4)	C2—C3—C23—C24	42.2 (4)
C6—C1—C7—C12	115.2 (3)	C4—C3—C23—C24	−138.6 (3)
C12—C7—C8—O2	177.8 (3)	C10—C9—C27—C30	−117.0 (4)
C1—C7—C8—O2	−7.5 (4)	C8—C9—C27—C30	61.7 (4)
C12—C7—C8—C9	−2.7 (4)	C10—C9—C27—C29	121.0 (3)
C1—C7—C8—C9	172.0 (3)	C8—C9—C27—C29	−60.3 (4)
C7—C8—C9—C10	1.6 (4)	C10—C9—C27—C28	1.8 (5)
O2—C8—C9—C10	−178.9 (3)	C8—C9—C27—C28	−179.4 (3)
C7—C8—C9—C27	−177.2 (3)	C12—C11—C31—C34'	87 (3)
O2—C8—C9—C27	2.3 (4)	C10—C11—C31—C34'	−94 (3)
C8—C9—C10—C11	−0.1 (5)	C12—C11—C31—C34	−99.1 (9)
C27—C9—C10—C11	178.7 (3)	C10—C11—C31—C34	79.3 (9)
C9—C10—C11—C12	−0.3 (5)	C12—C11—C31—C32'	−41.7 (14)
C9—C10—C11—C31	−178.8 (3)	C10—C11—C31—C32'	136.8 (14)
C10—C11—C12—C7	−0.8 (4)	C12—C11—C31—C33	138.9 (6)
C31—C11—C12—C7	177.8 (3)	C10—C11—C31—C33	−42.7 (7)
C8—C7—C12—C11	2.3 (4)	C12—C11—C31—C32	23.1 (8)
C1—C7—C12—C11	−172.6 (3)	C10—C11—C31—C32	−158.4 (7)
O4—S1—C13—C18	−175.4 (3)	C12—C11—C31—C33'	−154.5 (18)
O3—S1—C13—C18	−42.9 (3)	C10—C11—C31—C33'	23.9 (18)
O1—S1—C13—C18	68.6 (3)		

### Hydrogen-bond geometry (Å, º)

**Table:**

*D*—H···*A*	*D*—H	H···*A*	*D*···*A*	*D*—H···*A*
O2—H2*A*···O4	0.82	2.59	3.322 (4)	149
C24—H24*C*···O3^i^	0.96	2.67	3.372 (4)	130
C25—H25*C*···O4^ii^	0.96	2.64	3.500 (4)	150
C20—H20*B*···O3^ii^	0.96	2.69	3.609 (5)	160

Symmetry codes: (i) −*x*, −*y*, −*z*+1; (ii) −*x*+1, −*y*, −*z*+1.

## References

[bb1] Bruker (2004). *APEX2*, *SAINT* and *SADABS* Bruker AXS Inc., Madison, Wisconsin, USA.

[bb2] Sheldrick, G. M. (2008). *Acta Cryst.* A**64**, 112–122. 10.1107/S0108767307043930 18156677

[bb3] Wang, C. & Wu, J. (2012). *Acta Cryst.* E**68**, o93. 10.1107/S1600536811052664 PMC3254444 22259592

[bb4] Wu, J., Pan, X., Wang, L. & Yao, L. (2009). *Acta Cryst.* E**65**, o155. 10.1107/S1600536808042323 PMC2968069 21581613

[bb5] Wu, J., Yu, T.-L., Chen, C.-T. & Lin, C.-C. (2006). *Coord. Chem. Rev.* **250**, 602–626.

